# Efficacy and Safety of Adding Electrolysis Device to Standard Methods of Maintaining Oral Hygiene in Patients with Fixed Orthodontic Appliance

**DOI:** 10.3390/healthcare14111498

**Published:** 2026-05-28

**Authors:** Đurđina Čolić, Slobodan Janković, Milica Jovanović, Vladimir Ristić, Dragana Stanišić, Aleksandar Acović, Aleksandra Arnaut, Raša Mladenović, Marko Milosavljević

**Affiliations:** 1 Department of Dentistry, Faculty of Medical Sciences, University of Kragujevac, 34000 Kragujevac, Serbia; djurdjinacolic@gmail.com (Đ.Č.); vristic7@gmail.com (V.R.); stanisic92@yahoo.com (D.S.); dr.acovic115@gmail.com (A.A.); sandra11_92@yahoo.com (A.A.); rasa.mladenovic@med.pr.ac.rs (R.M.); drm.milosavljevic@yahoo.com (M.M.); 2 Department of Pharmacology and Toxicology, Faculty of Medical Sciences, University of Kragujevac, 34000 Kragujevac, Serbia; slobnera@gmail.com

**Keywords:** oral hygiene, fixed orthodontic appliance, oral health, electrolysis device, gingival inflammation, dental plaque

## Abstract

**Background/Objectives:** Fixed orthodontic appliances interfere with oral hygiene and contribute to plaque retention, gingival inflammation and demineralization of enamel. Standard techniques for keeping oral hygiene (tooth brushing, mouthwashes, dental floss, interdental brush, etc.) are not sufficiently effective. The aim of this study was to investigate the effectiveness, safety, tolerability, and influence on quality of life of an electrolysis device being added to standard techniques of oral hygiene in orthodontic patients, compared to standard methods only. **Methods:** This 6-month study was designed as an observational prospective-cohort investigation. Primary outcomes of the study were indices of gingival inflammation and bleeding, dental plaque indices, the number of white spots on enamel, and safety (incidence of adverse events). Secondary outcomes were quality of life and overall costs of keeping oral hygiene. **Results:** The addition of the Neo Pill device to standard oral hygiene maintenance measures was associated with improvements in oral health indices after 6 months; however, given the non-randomized, preference-driven design, these findings reflect an association and should not be interpreted as evidence of causal efficacy. After 6 months, the primary outcomes of the study were significantly reduced compared to the application of only standard oral hygiene methods (from 21 to 55% reduction); the quality of life related to oral health was higher (for 14%), the tolerability of maintaining oral hygiene was the same as with standard measures and the costs of maintaining oral hygiene consumables were lower in the Neo Pill group (median difference 30%); however, this figure excludes the acquisition cost of the device itself, which was donated to all participants by the manufacturer, and the 95% confidence interval for this difference includes zero. **Conclusions:** The addition of an electrolysis device to standard oral hygiene maintenance measures in people wearing fixed orthodontic appliances was associated with improvements in gingival inflammation, papillary bleeding, and dental plaque indices—outcomes measured with established clinical instruments. Apparent reductions in white-spot lesion counts were also observed but should be considered exploratory given the absence of calibrated or blinded lesion assessment. These findings are preliminary and do not establish causal efficacy.

## 1. Introduction

Fixed orthodontic appliances can pose a challenge to maintaining oral hygiene. The placement of fixed orthodontic appliances can compromise the patient’s oral environment through the presence of additional surfaces and impeding oral hygiene procedures, which can affect the oral microbiota balance [1]. Rough margins on the fixed orthodontic appliance can also result in microorganisms’ attachment, which can worsen oral hygiene conditions and cause gingivitis and further periodontitis [2].

Oral hygiene practices include brushing with toothpaste, flossing, using mouthwash, interdental brushes, and avoiding sweet foods and drinks [3]. Brushing should be done at least twice a day, using a soft-bristled toothbrush and a 45-degree angle to the gumline. Flossing or using an interdental brush should be done daily, to clean between the teeth and under the wires. Mouthwash can help reduce plaque and inflammation, but should not replace brushing and flossing. Sweet foods and drinks can increase the risk of tooth decay and enamel demineralization.

Oral hygiene status can be assessed by using different indices, such as the Orthodontic Plaque Index, the gingival index, and the Bleeding Index [4]. These indices measure the amount of plaque, inflammation, and bleeding in the gingival tissues around the orthodontic appliances. The oral hygiene status can also be visualized by using disclosing tablets or toothpaste, which stain the plaque and show where it needs to be removed.

Oral hygiene in patients with fixed orthodontic appliances is not satisfactory in the majority of cases. There is a significantly higher frequency of signs of gingival inflammation and amount of dental plaque in patients with fixed orthodontic appliances [5]. The gingival pocket depth and overall concentration of microorganisms are increased after wearing fixed orthodontic appliances [6]. Prolonged treatment increases the risk of plaque development around orthodontic brackets and bands. This can further lead to caries development and white-spot lesion development around fixed orthodontic brackets [7]. Among these devices, stainless steel lingual retainers, plain or braided, have much fewer periodontal complications than glass-fiber-reinforced retainers, but they still require intensive dental hygiene to prevent gingival inflammation, dental plaque and white spots on enamel [8].

Specialized devices aimed at improving oral hygiene through physical mechanisms other than conventional mechanical plaque removal are rarely described in the literature. One example is a blue-light-based device designed to reduce bacterial load in the oral cavity. Blue light of a specific wavelength and intensity has been reported to exert antimicrobial effects, primarily through photochemical reactions that may affect susceptible microorganisms within the biofilm environment. However, evidence regarding selective targeting of specific bacterial species and its impact on the overall oral microbiome remains limited. Although such devices are proposed to reduce oral bacterial burden, robust clinical evidence supporting selective antimicrobial action and sustained microbiome modulation is still insufficient [9]. Powered toothbrushes, including electric and sonic models, represent another category of adjunctive oral hygiene devices for orthodontic patients. Several randomized controlled trials and systematic reviews have reported that powered toothbrushes achieve superior plaque removal and gingival bleeding reduction compared to manual toothbrushes in patients with fixed orthodontic appliances, likely due to higher brushing frequency and consistent stroke mechanics. Sonic toothbrushes may additionally exert hydrodynamic forces that disrupt biofilm beyond direct contact. Nevertheless, their benefit is contingent on patient compliance and correct technique, and they do not fully compensate for the difficulty of cleaning interproximal and subgingival areas around brackets and archwires.

There is a great need for a simple medical device to maintain oral hygiene in an effective, safe and easy-to-use way. Recently, an innovation in this field has been made, and the Neo Pill electrolysis device was developed, which is proposed to cause detachment of bacteria from the fixed appliance and facilitate their elimination. Although the device was approved for sale and human use in Serbia, for wider use it is necessary to prove its efficacy and safety in clinical studies, which is exactly the intention of this project. If proved to be safe and effective, this device may enhance oral hygiene practices and contribute to the prevention of periodontal disease and caries. The aim of this study was to investigate the effectiveness, safety, tolerability, and influence on quality of life of the Neo Pill electrolysis device being added to standard techniques of oral hygiene in patients with fixed orthodontic appliances, and to make a comparison with standard techniques only (tooth brushing, dental floss, and mouthwash).

## 2. Materials and Methods

### 2.1. Study Design

**Figure 1:**
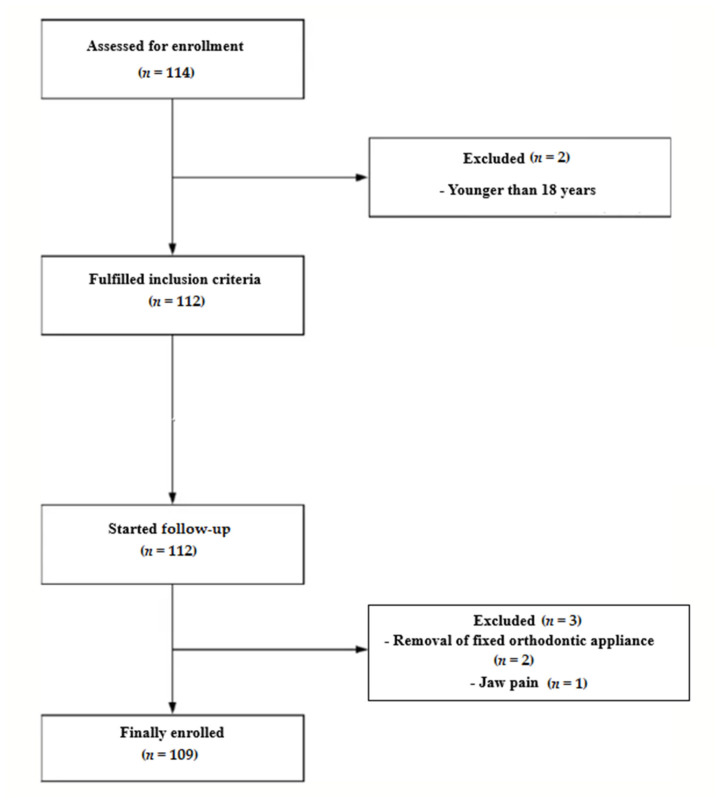
STROBE flowchart. STROBE, Strengthening the Reporting of Observational Studies in Epidemiology.

The research was designed as an observational prospective cohort study. Primary objectives were to investigate effectiveness, safety and tolerability of Neo Pill electrolysis device added to standard techniques of oral hygiene in patients with fixed orthodontic appliances, and to compare with standard techniques only. Secondary objectives were to investigate the influence of the Neo Pill electrolysis device on the quality of life and overall costs of keeping oral hygiene. The study was conducted from 1 July 2024 to 31 July 2025, at the Dentistry Department, Faculty of Medical Sciences, University of Kragujevac, Serbia. The study was endorsed by the Ethics Committee of the Faculty of Medical Sciences in Kragujevac, decision No 01-4098/6, dated 29 April 2024. The study was registered at ClinicalTrials.gov database, ID NCT06834139, and conducted following the STROBE guidelines (Strengthening the Reporting of Observational Studies in Epidemiology), as shown in a flow chart in Figure 1.

### 2.2. Design of Electrolysis Device

**Figure 2:**
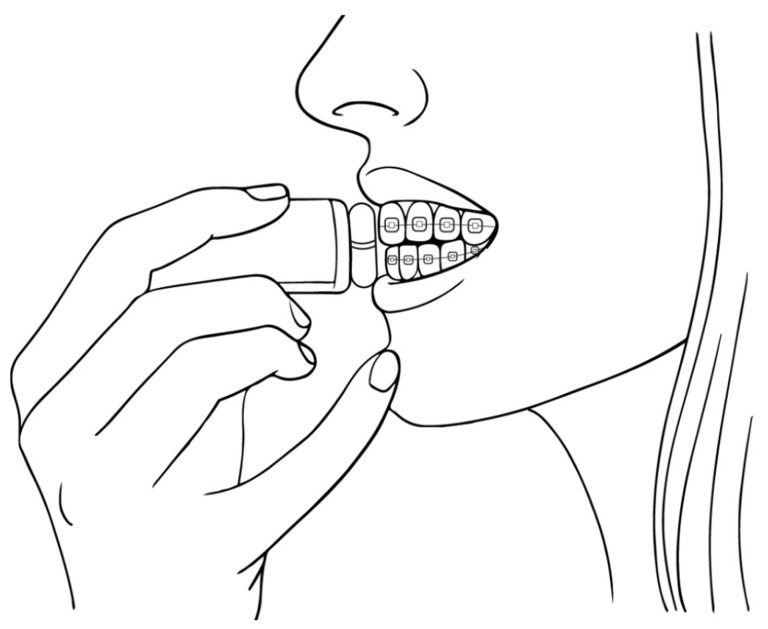
The Neo Pill device.

The Neo Pill electrolysis device (Figure 2), produced by Neofunction d.o.o., (Belgrade, Serbia), is positioned in contact with arcs of fixed orthodontic appliances and produces direct electric current (DC) with 3 V voltage, with a recommended duration of 30 s. Using the natural negative charge of bacterial surfaces, Neo Pill attracts and remove residual bacteria left after brushing. The Neo Pill device is made of Polilac PA757F NC plastic and brass alloy, with two AAA 1.5 V batteries within the enclosure. Metal surfaces that come into contact with an orthodontic appliance are covered by 24-carat gold. The Neo Pill device represents a pending patent with application number PCT/RS2024/000011 [10], and the current status is that the application has been published, pending further national/regional phase entry.

### 2.3. Inclusion and Exclusion Criteria

The inclusion criteria for the study were: provision of a signed and dated informed consent form, therapy with a fixed orthodontic appliance lasting at least 6 months, aged 18 years or above, good general health, permanent dentition, ability to maintain adequate oral hygiene, optimum dental health without immediate need for any allied dental procedure, and a fixed orthodontic appliance with a stainless steel arch. The exclusion criteria were: pregnancy, anticipated pregnancy, or breastfeeding; incarceration or institutionalized living; participation in another clinical study; acute and chronic systemic diseases; using antibiotics within the last 30 days; patients with pacemakers and other implanted devices that require a stable supply of electric energy; tooth decay; periodontitis; dental crowding; and a fixed orthodontic appliance with a nickel–titanium arch. The suspension criteria were: subject’s demand to discontinue the study, serious adverse events or unusual changes in clinical test results, and principal investigator’s decision to terminate the study (low rates of compliance, complications, or unable to sustain the study for various reasons).

### 2.4. Risk of Bias and Confounding

The findings of this study should be interpreted with consideration of potential sources of bias and confounding. Selection bias may be present due to the specific inclusion criteria and the characteristics of participants willing to use an additional oral hygiene device. Performance and compliance bias cannot be excluded, as patients using the electrolysis device may have been more motivated or adherent to oral hygiene practices overall. Furthermore, unmeasured confounding factors—such as dietary habits, baseline oral hygiene proficiency, or variations in orthodontic treatment duration—may have influenced the observed outcomes, although efforts were made to standardize procedures. Compliance bias is a further concern: because device use could not be objectively verified and relied entirely on patient self-report, the possibility that the Neo Pill group differed systematically from the standard hygiene group in overall hygiene engagement—independently of device use—cannot be excluded. Furthermore, the unequal group sizes (69 patients in the Neo Pill group versus 40 in the standard hygiene group) are themselves indicative of preference-driven allocation rather than random assignment, and may reflect systematic differences in patient motivation or dentist recommendation patterns that are not captured by the covariates included in the statistical models.

### 2.5. Management of the Patients

The study patients were advised about oral hygiene according to the preferences of their community care dentists independently from the study investigators. The Neo Pill electrolysis device was available on the Serbian market, and was recommended to patients according to the preferences of the community care dentists. The total duration of a patient’s participation in the study was 6 months. There were five study visits: V screen—screening visit; V0—baseline visit; V1—visit after one month; V2—visit after 3 months; and V3—visit after 6 months. Patients allocated to the Neo Pill group received standardized written and verbal instructions on device use at the baseline visit: the device was to be placed in contact with the archwire and buccal surfaces of the fixed orthodontic appliance for 30 s after each tooth-brushing session, twice daily. Patients were informed that the device produces a low-intensity direct current and were advised to discontinue use and report any discomfort. Adherence to the prescribed regimen was not verified by any objective method; the Neo Pill device does not incorporate usage logging or a timer, and no structured compliance diary was used. Compliance was assessed solely through informal patient self-report at scheduled follow-up visits, which is subject to recall and social desirability bias. All conclusions regarding device use, therefore, assume, but cannot confirm, adherence to the 30 s twice-daily protocol. It should be noted that background oral hygiene advice was provided by community care dentists independently from the study investigators, without a standardized protocol. As a result, the content and intensity of hygiene counseling may have differed systematically between patients who chose to use the Neo Pill device and those who did not. The linear mixed-effects models adjust for baseline outcome values, age, and sex; however, they do not fully account for unmeasured confounders such as motivation to comply with oral hygiene, dietary habits, or pre-existing oral hygiene proficiency. The statistical adjustment therefore reduces but cannot eliminate the influence of these factors on the observed between-group differences.

### 2.6. Study Outcomes

The primary outcomes of the study were the Turesky Modified Quigley Hein Plaque Index (TQHPI) [11] for both anterior and posterior teeth, the Gingival Inflammation Score (GIS) for both anterior and posterior teeth, the Papillary Bleeding Index (PBI) [12], the Ortho-Plaque Index (OPI) [13], the number of white spots on enamel (assessed by visual inspection by a single non-blinded investigator without formal calibration; results for this outcome should therefore be interpreted as exploratory), safety (incidence of adverse events), and tolerability (on Visual Analog Scale from 0 to 10, i.e., from minimum to maxi-mum tolerability). Secondary outcomes of the study were quality-of-life tolerability (on visual analogue scale (VAS) from 0 to 10, i.e., from the worst to the best quality of life) and costs of keeping oral hygiene (paid by the patients themselves).

### 2.7. Sample Size Calculation

In a study by Ren and associates [14], patients with orthodontic appliances who used mouthwashes with 0.12% chlorhexidine had a reduction in the gingival inflammation index by 0.45 points compared to placebo (standard deviation 0.25). If preventive treatment with an Electrolysis Apparatus is assumed to decrease gingival inflammation by 0.45 points, and assuming statistical power of 95% and a probability of type 1 error less than 0.05, we would need 20 patients in total, calculated by means of G*Power 3.1.9.7. software [15]. However, the study simultaneously evaluates seven primary efficacy outcomes—the Turesky Modified Quigley-Hein Plaque Index (TQHPI), the Gingival Inflammation Score (GIS), the Papillary Bleeding Index (PBI), the Ortho-Plaque Index (OPI), and white spot lesion counts for the upper jaw, lower jaw, and total—without correction for multiplicity. Testing multiple outcomes simultaneously without adjustment inflates the family-wise error rate (FWER), increasing the probability of at least one spurious statistically significant finding beyond the nominal α = 0.05. Applying the Bonferroni correction across the seven primary efficacy outcomes reduces the per-comparison significance threshold to α* = 0.05/7 ≈ 0.0071. Using this corrected threshold, retaining the same effect size (d = 1.80) and power (95%), and assuming a two-independent-samples design (Neo Pill + standard hygiene versus standard hygiene only), recalculation yields a minimum of 12 participants per group (24 in total). After adjusting for the observed dropout rate of 4.4%, the recommended minimum enrollment would be 26 participants in total.

A further source of multiplicity arises from the four repeated-measurement time points (baseline, one month, three months, and six months). When between-group comparisons for all seven efficacy outcomes are examined at each time point independently, the total number of between-group tests reaches 28 (7 outcomes × 4 time points), suggesting an even more conservative Bonferroni-corrected threshold of α* = 0.05/28 ≈ 0.0018 for any analysis examining individual outcome-by-visit combinations. Under this threshold, with the same effect size and 95% power, the minimum required sample size increases to 14 participants per group (28 total; approximately 30 after accounting for dropout). It should be noted that longitudinal repeated-measures models testing a single group-by-time interaction term for each outcome partially address this concern by reducing the effective number of independent tests compared with unadjusted pairwise comparisons across all visits. The enrolled and completed sample (*n* = 109) considerably exceeds the minimum requirement under any of the multiplicity-adjusted scenarios described above.

### 2.8. Statistical Analysis

The data were first described by measures of central tendency (mean and median) and variability (standard deviation and range). Normality of the data distribution was checked by the Kolmogorov–Smirnov test. The significance of differences in values of continuous variables among the repeated measurements was tested by Student’s *t*-test for dependent samples and one-way analysis of variance (when data distributions were normal) or by the Wilcoxon Signed Rank test and Friedman test (when data distributions were not normal). The significance of differences in values of continuous variables among the independent groups was tested by Student’s *t*-test for independent samples and one-way analysis of variance (when data distributions were normal) or by the Mann–Whitney U test and Kruskal–Wallis analysis of variance (when data distributions were not normal). The differences in values of categorical variables (e.g., frequencies) were tested by Chi-square or Fisher’s test. The above calculations were performed by Statistical Package for Social Sciences (SPSS) software, version 18.0 for Windows.

Additionally, linear mixed-effects models (REML estimation) were fitted for each outcome using the lme4-equivalent statsmodels implementation in Python version 3.14. The model specification for each outcome Y was

Y ~ Baseline + Group × Time + Age + Sex + (1 | SubjectID)

The baseline value of the respective outcome at V0 (baseline visit) was included as a covariate to adjust for pre-existing differences (ANCOVA approach). The treatment group was coded as Group (0 = standard hygiene only; 1 = Neo Pill + standard hygiene). Time was treated as a categorical factor with three levels (Visit1, Visit2, Visit3), with Visit1 (one-month visit) as the reference category. The Group × Time interaction tests whether the trajectory of change over time differs between groups. Age (continuous, years) and sex (0 = male, 1 = female) were included as potential confounders. A random intercept per subject was specified to account for within-person correlation across repeated measurements. All models converged successfully (*n* = 109 subjects, 327 observations per outcome). *p* values indicated statistical significance if *p* ˂ 0.05.

## 3. Results

**Figure 3:**
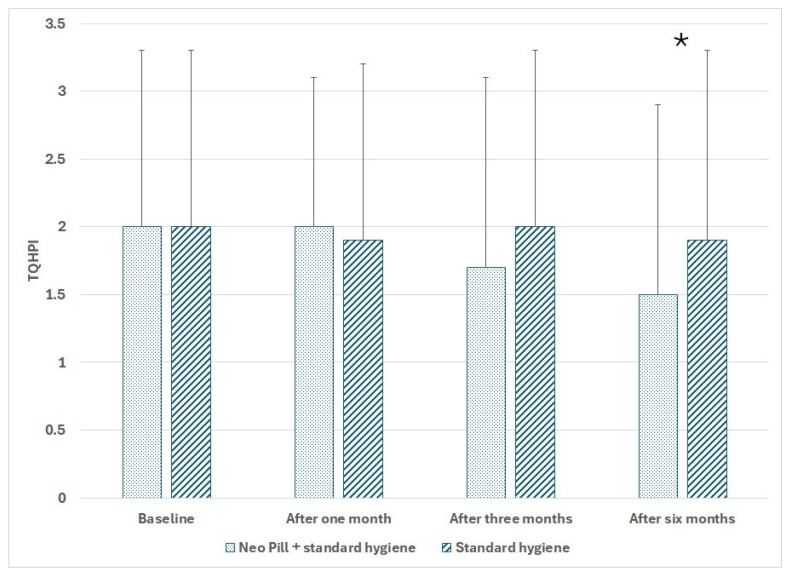
TQHPI values throughout the study for the Neo Pill + standard-oral hygiene and standard-oral hygiene-only groups. 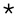
—Significant difference between the groups (*p* ≤ 0.05).

**Figure 4:**
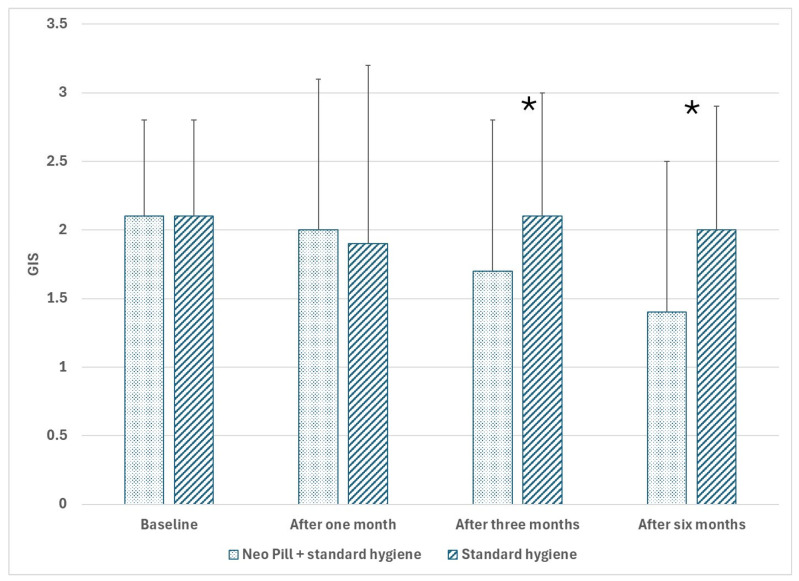
GIS values throughout the study for the Neo Pill + standard-oral hygiene and standard-oral hygiene-only groups. 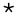
—Significant difference between the groups (*p* ≤ 0.05).

In total, 114 patients with orthodontic appliances took part in the study, and 5 of them were lost to follow-up. The screen/enrollment ratio was 95.6%, and the dropout ratio was 4.4%. Two patients dropped out of the study due to the removal of their fixed orthodontic appliance, two patients were younger than 18 years and one patient returned the electrolysis device due to jaw pain. Forty patients kept oral hygiene with standard methods, while sixty-nine patients in addition to these methods used the Neo Pill device. The age, sex and baseline values of oral hygiene markers per study group are shown in Table 1. Markers of oral hygiene and indices of tolerability, quality of life and treatment costs are shown for each visit in Table 2 and Figure 3 and Figure 4. Adding the Neo Pill to standard techniques of oral hygiene led to significant improvement in all of the study variables after 6 months, when compared to just standard techniques of oral hygiene. However, white-spot lesion counts—which showed the numerically largest between-group differences in both Table 2 and the mixed-effects model (Table 3)—were assessed by a single non-blinded, uncalibrated observer using visual criteria, and a net reduction in counts over six months is biologically difficult to interpret without quantitative confirmation; these findings should therefore be regarded as exploratory. No serious adverse events were observed throughout the study, both in the Neo Pill and standard techniques only groups.

Table 3 shows the results of the mixed-effects regression adjusted for baseline values and potential confounders. For oral health indices (TQHPI, GIS, PBI, OPI) and white-spot lesion counts, negative coefficients indicate greater improvement in the Neo Pill group. For tolerability and quality of life (VAS), positive coefficients indicate greater improvement in the Neo Pill group.

## 4. Discussion

This study showed that the addition of the Neo Pill device to standard oral hygiene maintenance measures in people wearing orthodontic appliances was associated with improvements in oral health after 6 months of use. After 6 months, gingival inflammation, papillary bleeding, and the presence of dental plaque were significantly reduced. White-spot lesion counts also showed between-group differences favoring the Neo Pill group; however, these findings require separate and stronger qualification: lesions were scored by a single non-blinded investigator without calibration, the measurement approach does not distinguish active from arrested lesions, and a net visual reduction in lesion count over six months is not readily explained by a mechanism of remineralization within this timeframe. These results are therefore considered exploratory and should not be accorded the same interpretive weight as the plaque and gingival indices. The quality of life related to oral health in the Neo Pill group was higher, and the tolerability of maintaining oral hygiene was the same as with standard measures. The costs of oral hygiene consumables were lower in the Neo Pill group by a median of approximately 30% at six months (700 vs. 1000 RSD); this figure is study-derived from participant-reported expenditure and is not a manufacturer claim. It must be noted, however, that the Neo Pill devices were donated to all participants by the manufacturer (Neofunction d.o.o., Belgrade, Serbia), meaning the device acquisition cost is entirely excluded from this comparison. In routine clinical practice, a patient purchasing the device commercially would need to offset its price against any consumable savings, and the net economic benefit would depend on the local retail price of the device. Furthermore, the 95% confidence interval for the between-group cost difference at 6 months includes zero, so the cost advantage should be regarded as suggestive rather than established.

The clinical magnitude of the 6-month between-group differences, expressed as Hodges–Lehmann location shifts, ranged from −0.40 to −0.50 index units for the plaque and gingival indices and from −1.0 to −3.0 lesions for white-spot counts. Standardized rank-biserial correlations ranged from 0.29 to 0.43, corresponding to small-to-medium effect sizes by conventional thresholds. Formally validated minimally important clinical differences (MIDs) for these indices in the orthodontic electrolysis context have not been established, so caution is warranted in translating these effect sizes into clinical recommendations. Notably, the 95% confidence interval for the upper-jaw white-spot lesion count included zero, suggesting that this particular comparison should be interpreted with additional caution. Among the secondary outcomes, quality of life showed the largest and most reliable effect (HL shift +1.00 VAS point; 95% CI +1.00 to +2.00; r = 0.46), while the confidence intervals for tolerability and treatment costs both included zero (HL shift +1.00, 95% CI 0.00 to +2.00, and −500 RSD, 95% CI −1000 to 0.00, respectively), suggesting that conclusions regarding these outcomes should be drawn with particular caution. The finding that tolerability did not differ significantly between groups is consistent with the interpretation that the Neo Pill device imposes no additional burden on patients, rather than conferring an advantage.

Fixed orthodontic appliances can affect the oral environment and microbiome by creating conditions that favor plaque accumulation and biofilm formation. At the same time, oral hygiene procedures are impeded. This can lead to increased concentrations of microorganisms in saliva and dental plaque, such as *Streptococcus mutans*, *Fusobacterium*, *Porphyromonas*, *Tannerella*, *Campylobacter*, and *Prevotella* [16]. The appliances can act as anchors for biofilm and plaque formation, leading to an accumulation of bacteria and other microorganisms in great amounts. This can cause dysbiosis, which manifests clinically as increased tooth decay and periodontitis. It should be noted, however, that the specific microbial species listed above were not directly assessed in the present study; our outcomes were limited to clinical indices of plaque and gingival health. The efficacy of manual toothbrushes, oral irrigators and dental flossing for keeping oral hygiene is decreased in patients with fixed orthodontic appliances [17]. Interdental brushes are somewhat more effective [18].

Adding chlorhexidine mouth rinse to the daily oral hygiene regimen reduced plaque and gingivitis development and was effective in improving the appearance of the gingiva in orthodontic patients [19]. The chlorhexidine varnish decreased the gingival overgrowth in patients undergoing orthodontic treatment, as well as the level of mutans streptococcus and lactobacillus colonies, and the development of white-spot lesions in patients with fixed orthodontic appliances [20]. The Miswak also proved to be effective in reducing dental biofilm and gingivitis. Miswak is a natural toothbrush made from the twigs of the Salvadora persica tree, which has been used for centuries in traditional oral hygiene practices [21].

However, fixed-orthodontic-appliance patients suffer limitations on the effective control of biofilm by mechanical methods, bringing the need for a coadjutant in the control of inflammation and oral health improvement. Additionally, compliance of the patients to the recommended schedule and techniques of tooth brushing, interdental brushing, dental floss, Miswak, or mouth rinsing could be problematic [22]. Patients self-reported largely consistent use of the Neo Pill device at follow-up visits; however, this was the sole means of compliance assessment. No objective verification was possible, as the device does not incorporate usage logging or timer functionality and no structured diary was employed. Consequently, it cannot be confirmed that all participants in the Neo Pill group adhered to the 30 s twice-daily protocol, and the magnitude of the observed between-group differences may partly reflect differences in general oral hygiene engagement rather than device use specifically. It is also worth noting that participation in a clinical study may itself alter patients’ oral hygiene behavior, as awareness of being monitored tends to increase motivation and adherence to hygiene instructions. Such behavioral modification may have contributed to improvements in both groups and should be considered when interpreting the observed differences.

The Neo Pill device, when administered, produces direct electrical current throughout the fixed orthodontic appliance and saliva. The following mechanistic explanations are hypothetical, as none of these processes were directly measured in the present study. DC causes migration of charged electrolytes (e.g., Na^+^, Cl^−^, HCO_3^−^_) in saliva, potentially altering local ionic concentrations and pH [23]. At sufficient current intensity, water electrolysis may occur, generating hydrogen, oxygen and probably reactive oxygen species [24]. Stimulation of parasympathetic nerve fibers by DC can increase the production of saliva [25]. Besides these effects that may promote the removal of microorganisms from the fixed orthodontic appliance, there is also a direct inhibitory action of DC on microbes, probably due to the formation of hypochlorous acid from sodium chloride and water [26]. Exposure of bacteria to an electric field with a strength of 2–10 V/m causes inhibition of their growth [27]. The study of Luo et al. [28] showed that DC alters the surface of phenol-degrading bacteria: an electric current of 20 mA increased the surface hydrophobicity and flattened the cell shape, and a higher current (40 mA) increased the surface extracellular substances and the net negative surface electrostatic charge. Administration of a direct current of 2–10 mA, with the voltage ranges 4–20 V, and with resistance ranging from 2 to 6 Ω, led to significant diminution of biofilm on dental implants [29]. It is hypothesized that the positive associations of the Neo Pill with oral health outcomes observed in this study may be related to the antibacterial properties of direct electrical current, but this remains to be confirmed by studies that directly measure microbiological and electrochemical parameters.

A special quality of the Neo Pill device is that no serious adverse events were recorded with Neo Pill use. Nevertheless, one participant discontinued use due to jaw pain, and non-serious device-related discomfort was not systematically collected across all participants. Therefore, a definitive safety conclusion cannot be drawn from a single observational study of this size, and safety should be formally evaluated in future controlled trials with pre-specified adverse event monitoring.

Due to the non-randomized observational study design, patients were not randomly allocated to intervention groups. The decision to use the Neo Pill device depended on routine clinical practice and patient preference, which introduces potential selection bias. Therefore, causal interferences should be interpreted cautiously, and the observed associations may partly reflect differences in motivation, compliance, or other unmeasured confounders.

**There are several limitations of our study**. First, and most centrally, the non-randomized observational design means that group membership was determined by patient and community dentist preferences rather than by random allocation. Background oral hygiene advice was delivered by community dentists independently from the study investigators, without standardization, so the content of standard care differed between groups in ways that were not measured or controlled. The group imbalance (69 Neo Pill users versus 40 non-users) further reflects this preference-driven allocation. Although the mixed-effects models adjust for baseline outcome values, age, and sex, they do not account for motivational differences, dietary habits, or baseline oral hygiene proficiency—confounders that the authors themselves identify as plausible—and therefore cannot eliminate confounding. All findings should be interpreted as describing an association between Neo Pill use and oral health outcomes, not as evidence of causal efficacy. Second, changes in the electrolyte composition of saliva and oral microbiome throughout the study were not followed, precluding inferences about mechanisms of improving oral health by the Neo Pill device. Also, no measurements of the intensity of the DC created by the Neo Pill in the oral cavity of the study subjects were conducted, which further obscures the mechanisms of the Neo Pill’s beneficial effects. White-spot lesion assessment represents a particular methodological weakness: lesions were evaluated by a single investigator using visual criteria only, without formal calibration exercises, without blinding to group allocation, and without quantitative methods such as quantitative light-induced fluorescence (QLF) or the International Caries Detection and Assessment System (ICDAS). This approach carries a substantial risk of both random measurement error and systematic observer bias. Furthermore, the biological plausibility of a net reduction in white-spot lesion counts over six months under these conditions is uncertain, since true remineralization to the point of visual disappearance is not reliably achievable within this timeframe without dedicated remineralizing agents. For these reasons, WSL findings from this study should be regarded as hypothesis-generating only, and future studies should employ calibrated, blinded, quantitative lesion assessment.

## 5. Conclusions

In conclusion, the addition of the Neo Pill device to standard oral hygiene maintenance measures in people wearing orthodontic appliances is associated with improvements in clinical oral health indices, including gingival inflammation, papillary bleeding, and dental plaque. Apparent reductions in white-spot lesion counts were also observed but are considered exploratory given the non-calibrated, non-blinded visual assessment method employed, and cannot be interpreted as confirmed remineralization. The quality of life was higher with the Neo Pill device, and oral hygiene costs were lower over 6 months. There is also the important caveat that device acquisition costs were not included in this comparison as devices were donated by the manufacturer, and the confidence interval for the cost difference includes zero. The device was generally well tolerated, with no serious adverse events. These findings address the study aim of evaluating the effectiveness, safety, tolerability, and quality-of-life impact of the electrolysis device as an adjunct to standard oral hygiene methods. However, the results are preliminary and hypothesis-generating, given the non-randomized observational design; they establish an association but do not prove causal efficacy. Randomized controlled trials with standardized comparator care, formal microbiological and electrochemical assessments, blinded outcome evaluation, and systematic adverse event monitoring are warranted before recommending routine clinical adoption of this device.

## Figures and Tables

**Table 1:** Characteristics of the study subjects at baseline.

Variable	Patients Using Neo Pill (*n* = 69)	Patients Not Using Neo Pill (*n* = 40)	*p* Value *
Age	22.0 [6.0]	22.0 [6.0]	0.902
Sex (m/f)	23/46 (33.3%/66.7%)	12/28 (30.0%/70.0%)	0.719
TQHPI	2.0 [1.3]	2.0 [1.3]	0.373
GIS	2.1 [0.7]	2.1 [0.7]	0.666
PBI	2.1 [0.7]	1.8 [0.3]	0.312
OPI	2.2 [1.2]	2.0 [1.2]	0.394
Number of white spots: upper jaw	3.0 [3.0]	1.0 [3.0]	0.163
Number of white spots: lower jaw	2.0 [3.0]	2.0 [3.0]	0.525
Number of white spots in total	5.0 [7.0]	3.5 [6.0]	0.280
Tolerability	6.0 [3.0]	6.0 [3.0]	0.741
VAS	6.0 [3.0]	6.0 [3.0]	0.717
Treatment costs	1700.0 [2000.0] RSD	2000.0 [2000.0] RSD	0.689

*p* value—Probability of null hypothesis; TQHPI—The Turesky Modified Quigley Hein Plaque Index; GIS—Gingival Inflammation Score; PBI—Papillary Bleeding Index; OPI—Ortho-Plaque Index; VAS—Visual Analog Scale; RSD—Republic of Serbia Dinar. *—Independent-Samples Mann–Whitney U Test for continuous variables and Chi-square test for categorical variables.

**Table 2:** Comparison of oral health markers between the groups exposed to Neo Pill + standard hygiene and only to standard hygiene.

Oral Health Marker	Group	Baseline	After One Month	After Three Months	After Six Months	Significance of Differences Between the Visits *	Effect Size at 6 Months: HL Shift (95% CI); r
**TQHPI**	Neo Pill + standard hygiene	2.0 [1.3]	2.0 [1.1]	1.7 [1.4]	1.5 [1.4]	0.000	-
	Standard hygiene	2.0 [1.3]	1.9 [1.3]	2.0 [1.3]	1.9 [1.4]	0.041	-
Significance of difference among the groups **		0.373	0.708	0.096	0.013	-	−0.50 (−0.70; −0.20); r = 0.29
**GIS**	Neo Pill + standard hygiene	2.1 [0.7]	2.0 [1.1]	1.7 [1.1]	1.4 [1.1]	0.000	-
	Standard hygiene	2.1 [0.7]	1.9 [1.3]	2.1 [0.9]	2.0 [0.9]	0.023	-
Significance of difference among the groups **		0.666	0.309	0.034	0.001	-	−0.50 (−0.70; −0.20); r = 0.38
**PBI**	Neo Pill + standard hygiene	2.1 [0.7]	1.6 [0.9]	1.5 [1.0]	1.2 [1.1]	0.000	-
	Standard hygiene	1.8 [0.3]	1.8 [1.0]	1.8 [0.9]	1.6 [0.9]	0.577	-
Significance of difference among the groups **		0.312	0.531	0.215	0.007	-	−0.40 (−0.60; −0.10); r = 0.31
**OPI**	Neo Pill + standard hygiene	2.2 [1.2]	2.0 [1.1]	1.7 [1.5]	1.4 [1.3]	0.000	-
	Standard hygiene	2.0 [1.2]	2.0 [1.2]	2.0 [1.3]	1.9 [1.3]	0.022	-
Significance of difference among the groups **		0.394	0.713	0.142	0.007	-	−0.50 (−0.70; −0.20); r = 0.31
**Number of white spots: upper jaw**	Neo Pill + standard hygiene	3.0 [3.0]	2.0 [3.0]	1.0 [4.0]	1.0 [3.0]	0.000	-
	Standard hygiene	1.0 [3.0]	2.0 [3.0]	2.0 [3.0]	2.0 [3.0]	0.000	-
Significance of difference among the groups **		0.163	0.539	0.245	0.003	-	−1.00 (−2.00; 0.00); r = 0.34
**Number of white spots: lower jaw**	Neo Pill + standard hygiene	2.0 [3.0]	2.0 [3.0]	2.0 [3.0]	1.0 [3.0]	0.000	-
	Standard hygiene	2.0 [3.0]	2.0 [3.0]	2.0 [3.0]	3.0 [3.0]	0.000	-
Significance of difference among the groups **		0.525	0.779	0.092	0.000	-	−1.00 (−2.00; −1.00); r = 0.43
**Number of white spots in total**	Neo Pill + standard hygiene	5.0 [7.0]	4.0 [7.0]	4.0 [6.0]	2.0 [6.0]	0.000	-
	Standard hygiene	3.5 [6.0]	3.5 [6.0]	4.0 [5.0]	4.5 [6.0]	0.000	-
Significance of difference among the groups **		0.280	0.625	0.124	0.000	-	−3.00 (−4.00; −1.00); r = 0.41
**Tolerability**	Neo Pill + standard hygiene	6.0 [3.0]	6.0 [3.0]	7.0 [3.0]	7.0 [3.0]	0.000	-
	Standard hygiene	6.0 [3.0]	6.5 [3.0]	6.5 [3.0]	7.0 [3.0]	0.009	-
Significance of difference among the groups **		0.741	0.524	0.142	0.013	-	+1.00 (0.00; +2.00); r = 0.28
**VAS**	Neo Pill + standard hygiene	6.0 [3.0]	7.0 [3.0]	8.0 [3.0]	8.0 [3.0]	0.000	-
	Standard hygiene	6.0 [3.0]	6.5 [3.0]	7.0 [3.0]	7.0 [4.0]	0.003	-
**Significance of difference among the groups**		0.717	0.166	0.043	0.000	-	+1.00 (+1.00; +2.00); r = 0.46
**Treatment costs**	Neo Pill + standard hygiene	1700.0 [2000.0] RSD	500.0 [1000.0] RSD	500.0 [1000.0] RSD	700.0 [1500.0] RSD	0.000	-
	Standard hygiene	2000.0 [2000.0] RSD	1000.0 [1375.0] RSD	1100.0 [2000.0] RSD	1000.0 [1500.0] RSD	0.000	-
Significance of difference among the groups **		0.689	0.216	0.009	0.005	-	−500 (−1000; 0.00) RSD; r = 0.32

*p* value—Probability of null hypothesis; * related-samples Friedman’s two-way analysis of variance by ranks; ** independent-samples Mann–Whitney U test. For the 6-month between-group comparisons, the Hodges–Lehmann (HL) location shift (Neo Pill minus Standard) and its 95% bootstrap confidence interval (5000 resamples) are reported as a measure of effect magnitude, together with the rank-biserial correlation (r) as a standardized effect size (|r| ≈ 0.1 small, ≈0.3 medium, ≈0.5 large). The 95% CI for white-spot lesions of the upper jaw includes zero, indicating that this specific comparison does not statistically exclude a null difference. Similarly, the 95% CIs for tolerability and treatment costs include zero at the 6-month visit, indicating that these differences should be interpreted with caution; the between-group difference in quality of life (VAS) was the most robust of the secondary outcomes, with a CI entirely above zero and a medium-to-large effect size (r = 0.46).

**Table 3:** Results of the mixed-effects regression adjusted for baseline values and potential confounders.

Outcome	Group Effect at Visit1 β (95% CI)	*p*	Group × Time Visit2 β (95% CI)	*p*	Group × Time Visit3 β (95% CI)	*p*	ICC
**Turesky Modified Quigley-Hein Plaque Index (TQHPI)**	−0.192 (−0.306; −0.078)	0.001	−0.217 (−0.298; −0.135)	<0.001	−0.388 (−0.470; −0.307)	<0.001	0.742
**Gingival Inflammation Score (GIS)**	−0.169 (−0.283; −0.054)	0.004	−0.166 (−0.267; −0.064)	0.001	−0.349 (−0.451; −0.247)	<0.001	0.604
**Papillary Bleeding Index (PBI)**	−0.198 (−0.311; −0.086)	0.001	−0.106 (−0.198; −0.013)	0.025	−0.297 (−0.389; −0.204)	<0.001	0.655
**Ortho-Plaque Index (OPI)**	−0.169 (−0.282; −0.056)	0.003	−0.189 (−0.275; −0.103)	<0.001	−0.389 (−0.475; −0.302)	<0.001	0.706
**White Spot Lesions—Upper Jaw (count)**	−0.270 (−0.588; 0.049)	0.097	−0.828 (−1.138; −0.518)	<0.001	−1.666 (−1.976; −1.356)	<0.001	0.520
**White Spot Lesions—Lower Jaw (count)**	−0.126 (−0.446; 0.194)	0.439	−0.807 (−1.109; −0.505)	<0.001	−1.688 (−1.990; −1.386)	<0.001	0.553
**White Spot Lesions—Total (count)**	−0.422 (−0.922; 0.078)	0.098	−1.621 (−2.069; −1.173)	<0.001	−3.339 (−3.787; −2.892)	<0.001	0.595
**Tolerability (VAS, 0–10)**	0.410 (0.062; 0.758)	0.021	0.285 (0.016; 0.554)	0.038	0.630 (0.361; 0.899)	<0.001	0.701
**Quality of Life (VAS, 0–10)**	0.664 (0.337; 0.992)	<0.001	0.261 (−0.014; 0.536)	0.063	0.963 (0.688; 1.239)	<0.001	0.645

β = Regression coefficient; 95% CI = 95% confidence interval; ICC = intraclass correlation coefficient. Bold values indicate *p* < 0.05. Group effect is estimated at V1 (reference visit). Group × Time V2 and V3 are interaction terms reflecting additional between-group divergence at 3 and 6 months relative to 1 month. Reference category: Group = 0 (standard hygiene), Time = V1 (1-month visit). White-spot lesion outcomes (upper jaw, lower jaw, total) show the largest model coefficients but were assessed by a single non-blinded, uncalibrated observer; their effect estimates carry a higher risk of measurement error than the plaque and gingival indices and should be interpreted as exploratory.

## Data Availability

The original contributions presented in this study are included in the article. Further inquiries can be directed to the corresponding author.

## Abbreviations

The following abbreviations are used in this manuscript:

DC	Direct Electric Current
TQHPI	Turesky Modified Quigley Hein Plaque Index
GIS	Gingival Inflammation Score
PBI	Papillary Bleeding Index
OPI	Ortho Plaque Index
VAS	Visual Analog Scale
RSD	Republic of Serbia Dinar
